# SARS-CoV-2 in Domestic UK Cats from Alpha to Omicron: Swab Surveillance and Case Reports

**DOI:** 10.3390/v15081769

**Published:** 2023-08-19

**Authors:** Sarah Jones, Grace B. Tyson, Richard J. Orton, Katherine Smollett, Federica Manna, Kirsty Kwok, Nicolás M. Suárez, Nicola Logan, Michael McDonald, Andrea Bowie, Ana Da Silva Filipe, Brian J. Willett, William Weir, Margaret J. Hosie

**Affiliations:** 1 MRC-University of Glasgow Centre for Virus Research, Glasgow G61 1QH, UK; g.tyson.1@research.gla.ac.uk (G.B.T.);; 2 School of Biodiversity, One Health and Veterinary Medicine, University of Glasgow, Glasgow G61 1QH, UKwillie.weir@glasgow.ac.uk (W.W.); 3 Bath Vet Referrals, Rosemary Lodge Veterinary Hospital, Wellsway, Bath BA2 5RL, UK

**Keywords:** SARS-CoV-2, COVID-19, coronavirus, pets, companion animals, reverse zoonosis, veterinary, animal welfare, feline, one health

## Abstract

Although domestic cats are susceptible to infection with SARS-CoV-2, the role of the virus in causing feline disease is less well defined. We conducted a large-scale study to identify SARS-CoV-2 infections in UK pet cats, using active and passive surveillance. Remnant feline respiratory swab samples, submitted for other pathogen testing between May 2021 and February 2023, were screened using RT-qPCR. In addition, we appealed to veterinarians for swab samples from cats suspected of having clinical SARS-CoV-2 infections. Bespoke testing for SARS-CoV-2 neutralising antibodies was also performed, on request, in suspected cases. One RT-qPCR-positive cat was identified by active surveillance (1/549, 0.18%), during the Delta wave (1/175, 0.57%). Passive surveillance detected one cat infected with the Alpha variant, and two of ten cats tested RT-qPCR-positive during the Delta wave. No cats tested RT-qPCR-positive after the emergence of Omicron BA.1 and its descendants although 374 were tested by active and eleven by passive surveillance. We describe four cases of SARS-CoV-2 infection in pet cats, identified by RT-qPCR and/or serology, that presented with a range of clinical signs, as well as their SARS-CoV-2 genome sequences. These cases demonstrate that, although uncommon in cats, a variety of clinical signs can occur.

## 1. Introduction

Many mammalian species, including domestic cats and captive big cats, are susceptible to SARS-CoV-2 infection by reverse zoonosis [1]. Infected domestic cats can transmit infection to other cats in experimental settings [2,3,4,5], whereas experimentally infected dogs are less likely to shed infectious virus [4,5]. Domestic cat-to-human transmission, with supportive viral sequencing data, has been reported [6], and in a zoo setting, lion-to-human transmission was concluded to have caused a cluster of human cases [7]. Consequently, the World Organisation for Animal Health (WOAH) has recommended surveillance of SARS-CoV-2 infections in animals [8].

The prevalence of SARS-CoV-2 in pet cats, detected by either RT-qPCR or by serology, was higher in households containing positive-testing humans, compared to households where pets were not known to have been exposed to humans with COVID-19 [9]; indeed, most studies reporting results of RT-qPCR testing of domestic cats have targeted COVID-19 affected households, which increases the probability of detecting cases. Early in the pandemic, between April and May 2020, 19 cats from COVID-19 households in the USA tested negative by RT-qPCR [10]. Conversely, 6/50 (12.0%) cats from COVID-19 households in Hong Kong tested positive between February and August 2020 [11], and 5/65 (7.7%) of cats from COVID-19 households in Canada were RT-qPCR positive in mid-2021 [12]. A later study conducted in Switzerland reported that 37/172 (21.5%) of cats from COVID-19 households tested between January 2021 and May 2022 were infected with SARS-CoV-2 [13]. The higher proportion of infected cats in the latter study coincided with the emergence of more contagious SARS-CoV-2 variants in the human population. The Alpha variant was estimated to be approximately 50% more transmissible between humans than the ancestral strain, with the Delta variant being in the order of 40 to 60% more transmissible than the Alpha variant [14].

Serological surveys have targeted pets from COVID-19 households while also testing wider feline populations. Positivity on serological tests persists for longer than RT-qPCR positivity [5], and so the former approach provides a more protracted window to obtain a positive test result from an animal which has been infected. Studies from Canada [12] and The Netherlands [9] both reported higher proportions of positive cats in COVID-19 households when testing for seropositivity (52% and 18.7%, respectively) compared to RT-qPCR positivity (7.7% and 3.9%, respectively).

A large-scale European survey reported seropositivity of 1.9% (19/1005) among feline samples taken early in the pandemic (January–July 2020) [15]. Brazil had larger numbers of feline cases early in the pandemic than many countries, with a seroprevalence of 7.3% reported in samples collected in 2020 [16]. A similar figure of 8.4% (12/143) was obtained from samples collected later (March–December 2021) in France [17]. In the UK, none of 96 cat serum samples tested between March and April 2020 possessed neutralising antibodies against SARS-CoV-2, whereas two of 90 (2.2%) collected in January 2021 were positive [18]. Samples submitted to the Veterinary Diagnostic Service (VDS) at the University of Glasgow between April 2020 and February 2022 showed an overall seroprevalence of neutralising antibodies of 3.2% [19]; the three-month period with the highest seroprevalence, 5.3% in September–November 2021, occurred during the Delta wave. Taken together, these studies indicate that the rate of SARS-CoV-2 infection in cats increased between the start of the pandemic and the Delta wave.

Numerous experimental [2,3,4,5,20] and natural [11,21,22] infections of domestic cats have been reported as subclinical or very mild. However, one study reported that 50% (n = 55) of infected cats showed clinical signs, with sneezing and lethargy being the most noted [23], and another reported respiratory and/or gastrointestinal signs in four of twelve cats infected with the Delta variant [24]. Additionally, a cat became severely ill when infected with SARS-CoV-2 in 2020, showing mainly respiratory signs [25]. Our group reported a case of an infected kitten which was euthanised as a result of severe respiratory disease. On post mortem examination, histopathological changes consistent with viral pneumonia were seen and SARS-CoV-2 nucleocapsid and RNA were detected in the lungs [26]. There have been other reports of respiratory disease in SARS-CoV-2-infected cats, including cases showing upper respiratory signs [27], pneumonia [28] and acute dyspnoea [29]. Additionally, an epidemiological link between the Alpha variant and myocarditis in dogs and cats was suspected [30] and severe SARS-CoV-2 associated disease with cardiac pathology was described in two cats [31,32]. Clinical infections with respiratory signs have also been reported in lions and tigers [33,34,35], and two tigers in two different locations were euthanised because of worsening respiratory signs (summarised in [36]).

Our previous study tested 387 oropharyngeal swabs from UK cats for the presence of SARS-CoV-2 RNA [26]. The swabs had been collected between March and July 2020 and submitted to the University of Glasgow VDS for testing for other feline respiratory pathogens. Only a single positive sample (0.23%) was detected, but, given the low human seroprevalence in the UK during this period, it was estimated that only 19 of these samples would have been from COVID-19 households. Similarly, another group reported a single positive RT-qPCR result amongst 260 cats presented to veterinary clinics in the first wave of the pandemic in Italy and Germany [37]; only six of those cats were thought to have been exposed.

Here, we report the findings of a combined passive and active surveillance programme conducted from 2021 to February 2023. We hypothesised that, as more infectious variants emerged and more people became infected following the relaxation of COVID-19 protective strategies, more cats would become infected with SARS-CoV-2. We also describe the clinical features of the SARS-CoV-2 infected cats that were identified during this period.

## 2. Materials and Methods

Respiratory swabs submitted dry or in viral transport medium (VTM) to VDS between June 2021 and February 2023 were tested for the presence of SARS-CoV-2 RNA in the active surveillance component of the study. These swabs had been submitted for routine respiratory pathogen testing which did not include testing for SARS-CoV-2. Most samples were taken from animals with clinical signs, with only a small proportion of swabs coming from healthy animals, for example for pre-breeding testing.

Dry swabs were placed in 1 mL VTM and agitated. Nucleic acid extraction was performed using 200 µL of VTM as the template for a Taco™ mini Automatic Nucleic Acid Extraction System machine (GeneReach, Taichung City, Taiwan), which was operated as per the manufacturer’s instructions. Nucleic acid extracts were kept frozen; for each sample, 5 µL of extract was used as template in a RT-qPCR assay for the detection of SARS-CoV-2 RNA, with primers targeting the N1 and N2 regions of the viral genome (further details in Appendix A: Supplementary methods (Appendix A.1, Table A1)). To confirm that novel variants could be detected using this RT-qPCR assay, RNA from cultured Alpha, Delta, Omicron BA.1, BA.2 and BA.5 SARS-CoV-2 virus was tested as these variants emerged.

Viral whole genome sequencing of every RT-qPCR positive sample was attempted. Due to the timeframe over which samples were sequenced, methods for sample preparation, sequencing and bioinformatic analysis were modified during the study. Whole genome sequencing was performed according to the ARTIC network nCoV-2019 (https://artic.network/ncov-2019, accessed on 14 May 2021) or using the EasySeq RC-PCR SARS-CoV-2 whole genome sequencing method (Nimagen, Nijmegen, Netherlands). Viral genome sequences obtained from cats were compared to the closest published human sequences. These methods are detailed in Appendix A: Supplementary methods (Appendix A.2 and Appendix A.3).

For the passive surveillance component of the study, we appealed to veterinary surgeons across the UK to submit swabs from animals suspected of having SARS-CoV-2 infections [38]. These swabs were processed in the same way, with one exception: dry swabs were added to 1 mL of L6 buffer [39] rather than VTM, to inactivate any SARS-CoV-2 present, and 200 µL of L6 was used for RNA extraction. For faecal swabs, polyvinylpolypyrrolidone (PVPP) was added to the L6 buffer. When requested, we performed bespoke serological testing using a pseudotype-based virus neutralisation assay (PVNT) to detect neutralising antibodies against the spike protein of SARS-CoV-2 B.1/D614G, Alpha, Delta and Omicron BA.1, as applicable, as described previously [19,40,41].

Sample metadata, which had been provided by veterinarians and owners, was extracted from the VDS laboratory information management system (LIMS). Results were stored and analysed in Excel (Microsoft, Redmond, Washington, USA). All positive swab results were reported to the UK Animal and Plant Health Agency (APHA) [42].

The VDS LIMS records of a separate set of patients, seropositive cats identified in a previous study [19], were also reviewed here. Those animals had been sampled between April 2020 and February 2022. Their clinical signs as described by submitting veterinarians, the reason for sampling, and the results of other tests are reviewed here.

Ethical approval was obtained from the University of Glasgow Veterinary Ethics Committee (EA27/20). Written consent was obtained from owners before investigating whether viral genome sequences were available from their samples.

## 3. Results

### 3.1. Active Surveillance

**Figure 1:**
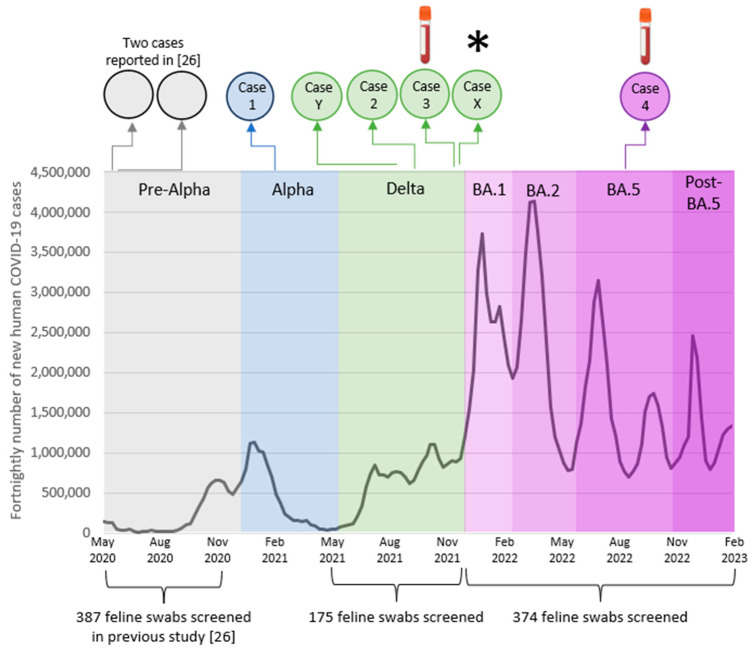
Line graph of the estimated fortnightly average number of people testing positive for SARS-CoV-2 in England, data from the Office for National Statistics (ONS) [26,43]. The timing of feline cases presented in this report is shown above the graph, and the sampling periods for the active surveillance component of this study are shown below it. The infected cat identified by active rather than passive surveillance (Cat X) is denoted by an asterisk (*) and the two cases which were positive on serology only (cases 3 and 4) are denoted by blood tube symbols.

A total of 549 oropharyngeal swabs that had been submitted for respiratory pathogen testing were screened for the presence of SARS-CoV-2 RNA. As only a small number of swabs was screened during the period of Alpha dominance, these were excluded from the study. We tested 175 swabs during the period when Delta was the dominant variant in humans, and 374 during Omicron dominance (Figure 1), of which 89 were tested when Omicron BA.1 dominated, 148 when BA.2 dominated, 91 when BA.5 dominated and 46 in the period post-BA.5 dominance.

A single swab from the Delta-dominant period tested positive by RT-qPCR (1/175, 0.57%), with Ct values of 22.2 for the N1 assay and 23.0 for the N2 assay (260,000–320,000 viral copies per µL RT-qPCR template). No positive samples were detected from the 374 swabs tested after the emergence of Omicron BA.1 and subsequent variants. The cat that tested positive during the Delta dominant period was a 14-year-old, female neutered (FN), domestic shorthair (DSH) (denoted Cat X to distinguish her from the cats from the four highlighted case reports, Table 1). The animal displayed mild respiratory signs of sneezing and nasal discharge and had shown only minimal improvement with antibiotic therapy. After amplicon sequencing, the complete SARS-CoV-2 genome was obtained and classified as Delta AY.4.2.1 lineage by PANGOLIN [44] and deposited in GISAID [45] (accession number EPI_ISL_17971927). There were no spike mutations not seen in closely related human-derived sequences (Table A3, Figure A3).

### 3.2. Passive Surveillance

We received one swab from a cat with suspected SARS-CoV-2 infection during the Alpha wave. This animal (Cat 1A) tested positive by RT-qPCR and further details are given below (case one). We received swab samples from ten animals where SARS-CoV-2 infection was a differential diagnosis during the Delta wave. One animal that tested RT-qPCR positive (Cat 2A) is detailed below (case two) and there was an additional weakly positive result (Cat Y, no associated detailed case report) (2/10, 20%). In the latter case only a small proportion of the SARS-CoV-2 genome was reconstructed after sequencing, which was insufficient to confirm the variant present (Appendix A.2.1, Appendix B). This nine-year-old, FN, Siamese cat (Cat Y) had nasal discharge and a history of recent exposure to SARS-CoV-2. Since BA.1 became dominant, samples from eleven cats suspected of being infected with SARS-CoV-2 were received (rectal/faecal only from one animal); none tested RT-qPCR positive (0/11, 0%).

### 3.3. Seropositive Cases Identified Previously

Seventy seropositive cats were detected in our active serosurveillance study, which was reported previously [19], and included animals sampled from April 2020 to February 2022. The information supplied by the submitting veterinarians, together with available diagnostic test results for the seventy seropositive cats identified in that study, are reviewed here.

No clinical histories were available for 22/70 seropositive animals. Nine animals had been sampled for routine disease screening, three for treatment monitoring and five following positive in-house tests for other pathogens. Thirty cats were reported to be unwell with a range of clinical signs; these included eight animals with gingivitis, five with pyrexia, three with ascites, three with ocular signs, three with neurological signs, one with tachypnoea and one with inappetence (data in Supplementary Materials). Tests requested by the submitting veterinarians included haematology, biochemistry, plasma proteins (albumin, globulin), A1-AGP (acute phase protein), T4, effusion analysis and tests for feline leukaemia virus, feline immunodeficiency virus, feline coronavirus, feline calicivirus, feline herpesvirus, *Toxoplasma gondii*, *Chlamydia felis* and *Mycoplasma felis*.

Of the 30 cats reported to be unwell at the time of sampling, other pathogens/diseases detected likely accounted for the reported clinical signs in eight cases, but in 22 cases they did not. Of these 22 cases, nine displayed clinical signs similar to those reported in people infected with SARS-CoV-2 (pyrexia, inappetence, conjunctivitis and tachypnoea), and eight had signs not, or only rarely, reported in humans with COVID-19, which were likely unrelated (polyuria, gingivitis, jaundice and increased appetite). In addition, two animals displayed neurological signs, one was anaemic, one had uveitis and one had collapsed.

### 3.4. Case Series

#### 3.4.1. Case One: Acute Pyrexia

**Figure A1:**
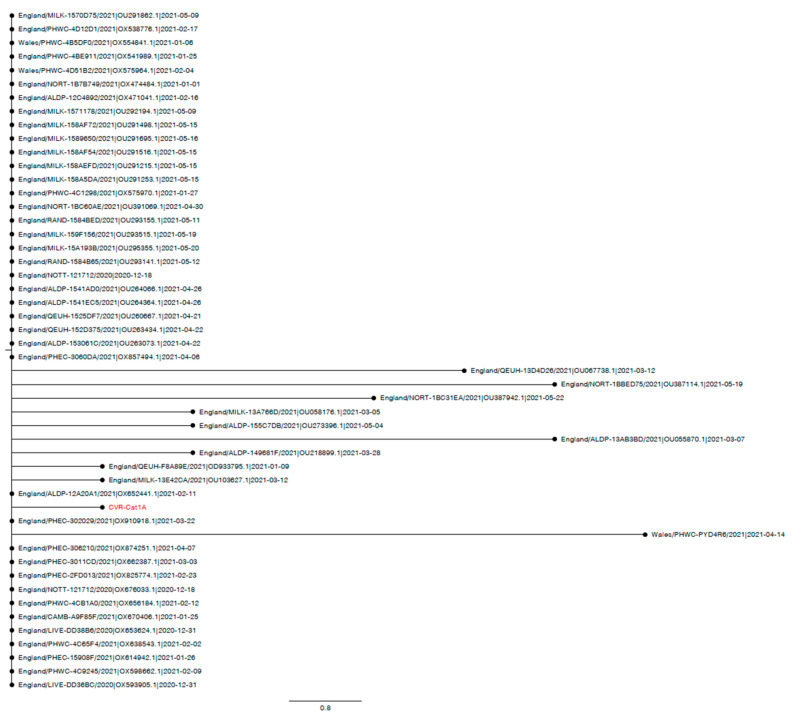
Cat 1A SARS-CoV-2 UShER phylogenetic local subtree using sequences from the public databases, with the cat sequence highlighted in red.

In February 2021, Cat 1A, a 9-month-old, male neutered (MN), DSH, developed anorexia and pyrexia for 24 h that resolved following treatment with a non-steroidal anti-inflammatory drug (NSAID). This illness occurred during a household COVID-19 outbreak, and the cat was suspected to have been infected with SARS-CoV-2. Three of four humans in the household tested positive for SARS-CoV-2 RNA by RT-qPCR (samples not sequenced). A nasal/oral swab from this cat tested positive in our SARS-CoV-2 N1 and N2 RT-qPCR assays (Ct 31; 200 viral copies per µL RT-qPCR template) and the complete viral genome was reconstructed from the sequence data (GISAID accession number EPI_ISL_17971924), with PANGOLIN classifying the sequence as an Alpha lineage. The sequence contained one synonymous spike mutation, L821L, not previously detected in any closely related human-derived viral sequences (Table A3, Figure A1). Blood samples were available from this cat, his two siblings and the two adult dogs in the household in May 2021. None of the other four animals had shown clinical signs. Cat 1A and another FN cat (Cat 1B) tested positive for neutralising antibodies; the highest titres were against the Alpha pseudotype (titres of Cat 1A: B.1/D614G 209, Alpha 1415, Delta ≤ 50 and of Cat 1B: B.1/D614G 193, Alpha 515, Delta 86). Neutralising antibodies were not detected in the samples from the other cat or the two dogs in the household.

#### 3.4.2. Case Two: Sudden Death

Cat 2A was an eight-year-old, MN, DSH with no known health problems, owned by the same household since kittenhood. The household also contained another MN DSH (Cat 2B) who was eleven years old.

**Figure 2:**
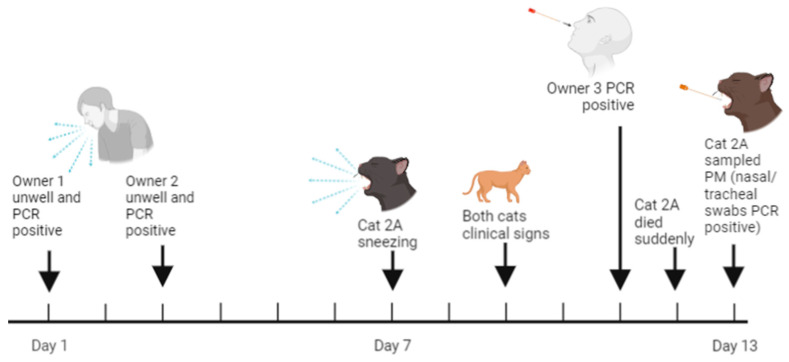
Timeline of the infection of Cat 2A. Created with BioRender.com.

All three people in the household developed COVID-19, testing positive by RT-qPCR of nasopharyngeal swab samples in October 2021 (samples not sequenced). During their ten-day isolation, both cats developed mild respiratory signs including sneezing (Figure 2). The sneezing abated, leaving both cats with mild epiphora and Cat 2B frequently licking its lips. Five days after the sneezing was most frequent, Cat 2A made an unusual sound, yawned and died suddenly.

**Figure A2:**
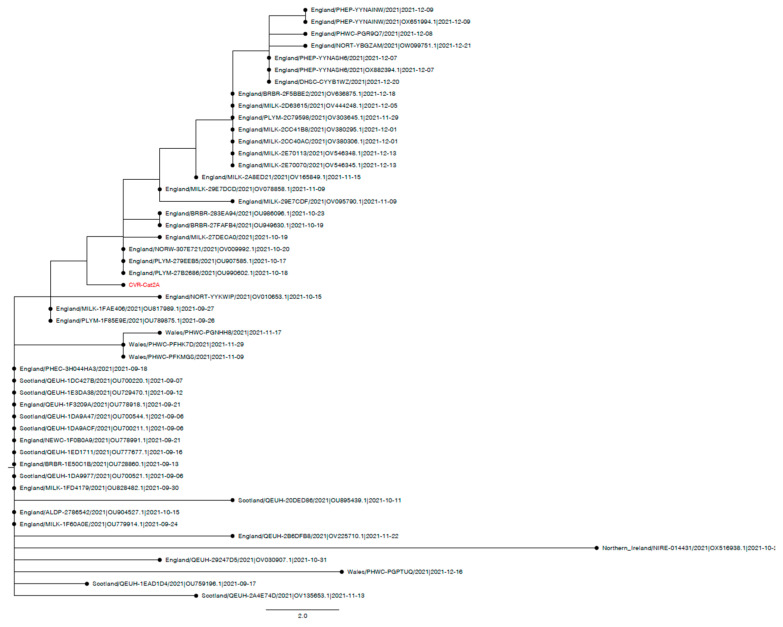
Cat 2A SARS-CoV-2 UShER phylogenetic local subtree using sequences from the public databases, with the cat sequence highlighted in red.

**Figure A3:**
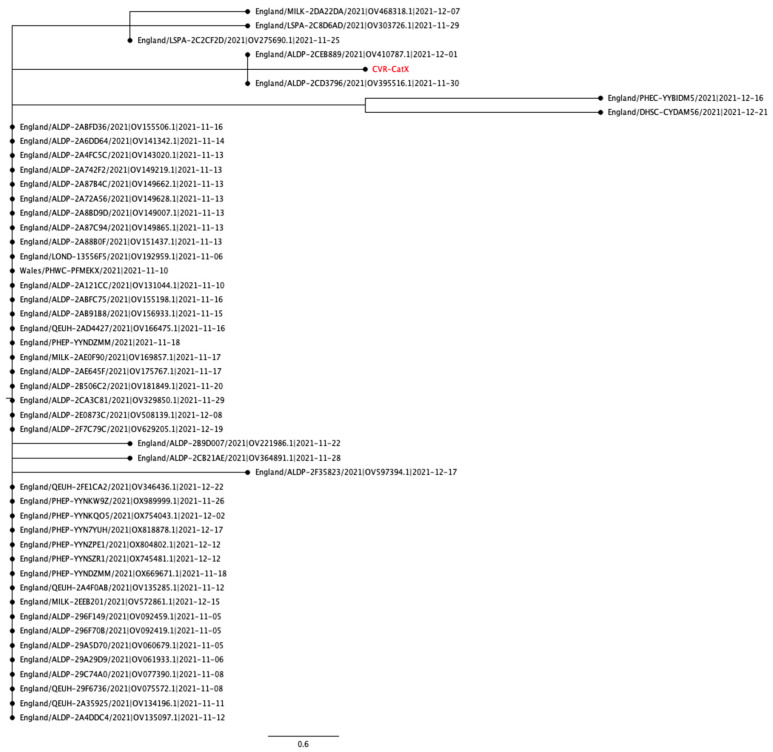
Cat X SARS-CoV-2 UShER phylogenetic local subtree using sequences from the public databases, with the cat sequence highlighted in red.

Dry swab samples were collected approximately 15 h post mortem from the nose, oral cavity, trachea and rectum of Cat 2A. Both the SARS-CoV-2 N1 and N2 RT-qPCR assays tested positive for one or more swabs. Ct values of between 17 and 19 (9.5–16.5 million copies per µL of template) for both assays were obtained from the nasal and tracheal swabs. The Ct was 37.5 (16 copies per µL, which was close to the limit of detection [approximately five copies not including RT step]) for N2 for the rectal swab, with no target detected using N1 primers, and no viral RNA was detected from the oral swab (Table A2). A near complete viral genome was reconstructed from the sequence data, except for one 485-nucleotide section due to a failed amplicon. The variant was classified as belonging to the Delta AY.4 lineage by PANGOLIN (GISAID accession EPI_ISL_17971926) and contained no novel spike mutations when the sequence was compared to the most closely related human-derived sequences (Table A3, Figure A2).

Cat 2B continued to show excessive lip licking for several weeks. At a veterinary examination 2.5 weeks after Cat 2A’s death, no abnormalities were detected. A blood sample was collected and neutralising antibody titres of 62, 57 and 1019 were measured for the B.1/D614G, Alpha and Delta variants, respectively, indicating that this cat had been infected with the Delta variant.

#### 3.4.3. Case Three: Anorexia

**Figure 3:**
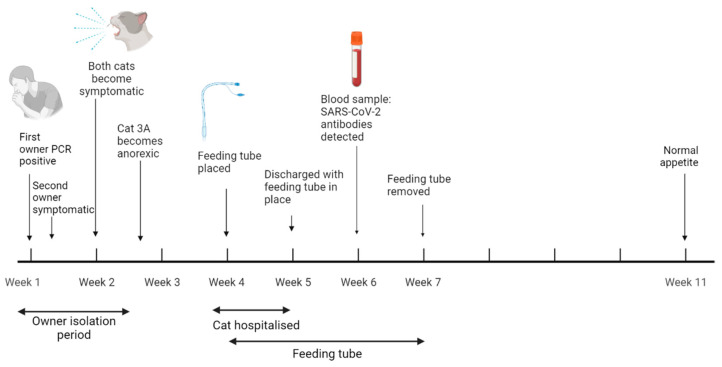
Timeline for Cat 3A. Created with BioRender.com.

Cat 3A was an eight-year-old, MN, Ragdoll cat. The household had owned Cat 3A and his full brother, Cat 3B (MN), since kittenhood; the cats had no medical issues. The patient’s owners both tested positive for SARS-CoV-2 by RT-qPCR of nasopharyngeal swabs in November 2021 (samples not sequenced). Towards the end of their isolation period, both cats became lethargic, listless, had watery ocular discharge and were sneezing (Figure 3). Cat 3B recovered uneventfully.

After the ocular/respiratory signs resolved, Cat 3A was presented to his usual veterinary practice with a primary complaint of three days of inappetence. Lethargy, mild vomiting of froth and chewing of plants had been observed by the owners. Clinical examination was unremarkable and an anti-emetic (maropitant, Vetemex, 1 mg/kg SC) and an H_2_ receptor antagonist (famotidine, 5 mg bid PO) were prescribed.

Four days later the patient re-presented at the practice having still not eaten. He had been noted to drink small amounts and had mild occasional vomiting and diarrhoea. Clinical examination revealed a tense abdomen and weight loss of 340g since the first consultation. The patient was admitted, and he remained in hospital for a week. Throughout this time the cat showed no desire to eat.

Haematology was unremarkable and serum biochemistry/electrolytes were normal, other than a mildly raised amylase and hypochloraemia. Pancreatic lipase immunoreactivity was normal and unsupportive of a diagnosis of pancreatitis. Intravenous fluid therapy was administered and was stopped after urine specific gravity indicated good hydration. Abdominal radiography and ultrasonography were largely unremarkable; areas of gastric wall close to the pylorus appeared thicker on the first ultrasonographic examination but normal on a subsequent scan four days later.

Whilst hospitalised, the patient was administered the anti-emetic, anti-nausea drugs maropitant and metoclopramide, and H_2_ receptor antagonist omeprazole. Mirtazapine was used as an appetite stimulant throughout the hospitalisation period and a single dose of vitamin B was given. Mild discomfort on abdominal palpation was noted on day two, but not at other times. Analgesia was provided in the form of buprenorphine and meloxicam (Table 2).

A nasogastric feeding tube was placed on day two of hospitalisation and was well tolerated by the patient. It was kept in place throughout the patient’s hospital stay; he was discharged with it in place and the owners were trained to administer tube feeds. The patient was discharged on metoclopramide and amoxycillin-clavulanate, the latter administered due to increased, harsh respiratory sounds.

Once back at home, Cat 3A was noted to behave differently towards his owners and sibling, appearing not to recognise them; he was wary of his sibling, with whom he had previously slept and engaged in mutual grooming. From a week post-discharge onwards, Cat 3A began to resume more typical social behaviour with Cat 3B. The feeding tube was removed two weeks post-discharge, by which point the cat was eating approximately 25% of his normal food intake. A week later his food intake had increased to 65–75% of normal, at which time all medications were stopped, and by a further month later his intake was considered normal.

No swab samples were available from Cat 3A. Blood was collected close to the date of hospital discharge and neutralising antibody titres of 286, 198, 1424 and 55 were detected to the B1/D614G, Alpha, Delta and Omicron BA.1 variants, respectively, consistent with infection with the Delta variant.

#### 3.4.4. Case Four: Respiratory Distress and Renal Disease

This household consisted of one person and two indoor-only cats. The person became unwell in September 2022 and tested positive on a COVID-19 lateral flow test. They made efforts to avoid passing infection onto their cats, such as not letting the cats on the bed or stroking them. Cat 4A, a 16-year-old, FN, domestic long-hair, had a history of gastrointestinal signs and received prednisolone (0.5 mg/kg PO sid) for presumed inflammatory bowel disease or small cell lymphoma.

Approximately ten days after the owner first tested positive, clinical signs were noticed in Cat 4A and included bilateral epiphora, coughing, lip licking, upper respiratory tract noise, reverse sneezing and exaggerated swallowing. The following day the cat was taken to her usual veterinary practice. Haematology and serum biochemistry showed mild changes mostly consistent with the cat’s underlying medical issues and treatment. The cat was treated for presumed ‘cat flu’ with bromhexine hydrochloride (0.1mg/kg PO sid, Bisolvon oral power, Boehringer Ingelheim, Bracknell, UK).

Twenty-four hours later, the cat was taken to an emergency veterinary service as her condition had worsened, with increased lethargy, inappetence, difficulty swallowing and persistent reverse sneezing. Dexamethasone (0.08 mg/kg IV) was administered. The next day, she was examined at a referral hospital under sedation and increased upper respiratory sounds and lack of airflow through the left nostril were noted. The patient received fluid therapy and oxygen.

Computed tomography (CT) was performed under a general anaesthetic. This revealed a well delineated, partially obstructive nasopharyngeal mass measuring 0.9 × 0.5 × 1 cm. There was also mild, generalised bronchial wall thickening consistent with age, asthma, bronchitis or bronchopneumonia. Sampling of the nasopharyngeal mass led to the diagnosis of an abscess with dysplastic cells. *Pasteurella multocida* infection was diagnosed and treated with amoxycillin and clavulanic acid (Clavaseptin 50 mg, Vetoquinol, Towcester, UK) for eight weeks; the patient recovered from the respiratory disease during this time but experienced an episode of polyuria/polydipsia, haematuria and stranguria. A month later the polyuria/polydipsia was still present, and the patient had lost weight and was azotaemic. She was diagnosed with Stage 2 Kidney Disease by IRIS staging [46]. Additionally, corticosteroid therapy was discontinued, and a hydrolysed diet supplied. There was no recurrence of intestinal signs following this change, making inflammatory bowel disease the most likely cause of the cat’s previous intestinal signs, rather than lymphoma.

SARS-CoV-2 RNA was not detected in a swab sample taken on day seven of the clinical signs. The patient showed neutralising antibody titres of 79, ≤50, ≤50 and 155 to SARS-CoV-2 variants B.1/D614G, Alpha, Delta and Omicron BA.1, respectively.

## 4. Discussion

Less than 1% of all swabs tested in the active surveillance part of this project were found to be positive. It was higher during the Delta wave (0.57%) compared to the first year of the pandemic (0.26% [26]). Additionally, 20% of swabs submitted specifically for SARS-CoV-2 testing were positive during the Delta wave. Although the number of positives was low, making it difficult to draw conclusions, an increase in test positivity had been expected, given the greater proportion of the UK human population infected during the Delta wave compared to earlier waves (Figure 1), as a result of the greater transmissibility of Delta compared to previous variants. This finding is consistent with the higher seroprevalence in animals reported in 2021 compared to 2020 in the UK [18] as well as the results of a study that identified several feline cases in SARS-CoV-2-positive households during the Delta wave in Switzerland [24].

A survey conducted by the Office for National Statistics (ONS) [43] estimated that the following percentages of the UK human population were infected when each variant was most common: pre-Alpha 7.0% (26 April–7 December 2020), Alpha 8.1% (8 December 2020–17 May 2021), Delta 24.2% (18 May–13 December 2021), BA.1 33.6% (14 December 2021–21 February 2022), BA.2 43.6% (22 February–6 June 2022) and BA.4/5 46.5% (7 June–11 November 2022). Given the large number of people infected since the emergence of Omicron BA.1, greater numbers of infected cats were predicted during this period compared to earlier periods. However, no RT-qPCR positive feline samples were detected despite screening 385 swabs across both sampling methods after the emergence of Omicron BA.1. This concurs with our serosurveillance data; BA.1 dominant antibody profiles were not observed when this variant displaced Delta [19].

Following an appeal to veterinary surgeons for samples from potential cases of SARS-CoV-2 infection, in line with the APHA case definition and guidelines [42], we accepted swabs for diagnostic testing. Early in the pandemic there were national protective measures and widespread human testing in place to reduce human-to-human transmission. This meant that people with current or recent household infections should not visit a veterinary surgeon unless there was an emergency. Consequently, the samples tested during this period were likely biased towards non-COVID-19 households and this could have accounted for the low number of positive swabs detected during the early waves of the pandemic. More recently, with the lifting of restrictions and the downturn in human testing, the likelihood of cats from infected households being taken to visit a veterinary surgery has increased. Despite these changed circumstances, no increase in feline SARS-CoV-2 infection prevalence was observed.

Cats experimentally challenged with Omicron BA.1.1 (B.1.1.529) were reported to remain sub-clinically infected, whereas cats challenged with B.1/D614G or Delta B.1.617.2 developed lethargy and pyrexia [47]. Likewise, seven naturally Omicron infected, RT-qPCR positive cats were reported to show no clinical signs [48]. While a proportion of the swabs submitted to VDS were collected from healthy animals, for example for pre-breeding screening, most were from cats with clinical signs of ocular, respiratory or oral disease. It may be hypothesised that if Omicron-infected cats do not, in general, show clinical signs, they will rarely be sampled for diagnostic testing. Cat 4A, which displayed an Omicron-dominant antibody profile, had an atypical, severe presentation of respiratory distress. Before presenting with respiratory disease, this cat had been administered daily corticosteroids for bowel disease, which could have made her more susceptible to SARS-CoV-2 infection. It has also been reported that Omicron-inoculated cats shed less virus and have lower levels of neutralising antibodies than B.1/D614G- or Delta-inoculated cats [47]. It is therefore possible that when cats are, or have been, infected with Omicron, there is less evidence of infection than was the case with earlier variants. Lastly, cats may be less susceptible to contracting Omicron than earlier variants.

Where humans in a household had been tested by RT-qPCR and sequence data were obtained from their cat, we attempted to obtain sequence data from the human samples; only a proportion of positive swab samples from humans in the UK were sequenced by public health bodies during most of our study period. Unfortunately, there were no sequence data for humans in our case households, which precluded comparison of viral sequences from linked human and feline cases. When our feline-derived viral sequences were compared to published human-derived viral sequences, only one cat had a single, synonymous spike mutation not seen in human samples (Table A3). This suggests that the virus is, at least initially, well conserved following human-to-cat reverse zoonosis, presumably maintaining the likelihood of onwards transmission from cats to humans, a phenomenon which has been reported [6]. In case 2, the chronology of SARS-CoV-2 diagnostic detection in the household was human, human, cat, cat and finally human. It is therefore possible that the virus could have been transmitted from cat-to-human, but this can neither be confirmed nor refuted with the data available. It remains unclear whether sustained cat-to-cat transmission, or sustained transmission between cats and other non-human species, could lead to novel viral mutations or variants, as reported in white-tailed deer [49] and mink [50].

Many of the seropositive animals identified by our serosurveillance [19] showed no clinical signs, mild clinical signs or had signs likely unrelated to SARS-CoV-2 infection at the time of sampling. This finding was consistent with numerous reports of mild or subclinical feline infections [2,3,4,5,9,11,20,21,22,23]. Additionally, seropositive animals might have experienced historical infections. However, in this study we also detected SARS-CoV-2 infections in animals with more severe disease, including individuals who suffered prolonged anorexia (Cat 3A), severe respiratory disease (Cat 4A) and sudden death (Cat 2A).

Of the four household cases described in detail, two of the cases (Cats 1A and 2A) tested positive by RT-qPCR, and viral sequencing confirmed the presence of SARS-CoV-2 variants that were circulating widely at the time of their illnesses. Cats in both households were seropositive, with the highest SARS-CoV-2 neutralising antibody titres evident against the variants detected in swabs. No swabs were tested from Cat 3A as it was assumed the timeframe for obtaining a positive SARS-CoV-2 RNA result had passed. However, this cat displayed a high neutralising antibody titre against the Delta variant, which was consistent with his illness and his owner’s positive tests occurring during the Delta wave. Cat 4A tested negative by RT-qPCR but showed the highest neutralising antibody titre against Omicron BA.1, consistent with infection with the Omicron variant which dominated at the time of the owner’s illness and the cat’s respiratory signs. This cat may have tested positive for SARS-CoV-2 RNA if sampled earlier or, alternatively, might not have shed detectable levels of virus. Experimental studies have shown that cats shed the ancestral variant for approximately five days [5], and field cases with longer shedding periods were identified during the pre-Alpha and Alpha waves [9,22]. However, a more recent study [48] reported that cats naturally infected with Omicron were PCR-positive on only the first day of four to five days of consecutive sampling, shedding low levels of virus.

A combination of anorexia, hyporexia and inappetence is a common and non-specific presentation of illness in cats. Underlying causes and mechanisms include pyrexia, pain, inflammation, neoplasia and diseases of multiple organs or systems [51]. Many potential causes of Cat 3A’s inappetence were excluded by diagnostic testing and imaging and no definitive diagnosis was reached. The timeline of Cat 3A’s illness is consistent with SARS-CoV-2 infection being the underlying cause. Potential mechanisms include gastrointestinal infection, nausea and a lost or altered sense of smell and taste. The anorexia continued long after the diarrhoea had resolved, suggesting nausea and a lost or altered sense of smell were more likely at that stage; loss of smell and/or taste is a recognised symptom in SARS-CoV-2-infected humans. Nausea might also explain the lip licking of Cat 2B which took weeks to resolve. Anosmia has been documented to persist months after initial infection and to reduce appetite and cause weight loss in humans [52], and therefore might explain the prolonged anorexia of Cat 3A. It was reported that a working dog with neutralising antibodies to SARS-CoV-2 had suspected anosmia, suggesting anosmia might affect non-human species [53]. Anosmia could also explain Cat 3A’s altered behaviour towards his sibling and owners. Indeed, SARS-CoV-2-infected people have reported a loss of intimacy with their partner due to not being able to detect their usual, natural scent [52].

As well as reporting lack of smell and/or taste, affected people have also reported that food smells disgusting and that they smell things that are not present, a phenomenon known as phantosmia. Once these clinical signs occur there is a possibility that learned food aversions will develop because the patient associates eating or food with bad smells or nausea [51]. Cat 3A was tube fed, which allows nourishment without coaxing or syringe feeding, both of which could induce secondary food aversion.

Unfortunately, a broad surveillance approach, such as the one taken in this study, cannot definitively demonstrate a causal link between the clinical signs reported and SARS-CoV-2 infection. No post mortem examination or tissue sampling of the cat (2A) that died suddenly were undertaken, so it is unknown whether cardiac lesions indicative of SARS-CoV-2 infection, or another disease process, were present. It was reported previously that a SARS-CoV-2-infected cat developed severe respiratory distress and thrombocytopaenia during a household infection, but it was concluded that an unrelated, pre-existing cardiomyopathy caused the observed pathology [54]. Here, RT-qPCR testing demonstrated that Cat 2A, a previously healthy middle-aged cat, was infected with SARS-CoV-2, which could cause, or exacerbate, cardiac pathology in cats [30,31,32]. SARS-CoV-2 related myocarditis and pneumonia was described in a cat with hypertrophic cardiomyopathy (HCM) [32], and sudden cardiac death in humans who appeared to be recovering from COVID-19 has been reported [55].

In addition, it was reported that a cat with a cardiac murmur developed severe and progressive respiratory disease after being exposed to SARS-CoV-2 [31]. Following a post mortem investigation, high loads of viral RNA and infectious virus were detected in the upper and lower respiratory tract and heart, and viral RNA was also detected in other organs. There was acute myocardial degeneration and necrosis, and viral particles were visualised in heart tissue, supporting a diagnosis of viral myocarditis. Pre-existing HCM and a pleural effusion were noted; the authors suggested that HCM, which can be subclinical, could be a risk factor for severe clinical signs in cats with SARS-CoV-2 infection due to the overexpression of ACE2 in HCM hearts.

Cat 4A developed respiratory distress following infection with SARS-CoV-2. This is consistent with other reports of SARS-CoV-2-induced respiratory pathology in cats and with imaging of Cat 4A revealing lower airway pathology. Cats with SARS-CoV-2-associated respiratory disease are mostly reported to have mild clinical signs, but more severe presentations have also been reported, including laboured breathing [25], pneumonia [28] and acute dyspnoea with air hunger and rales [29]. Cat 4A was later diagnosed with a bacterial upper airway infection and abscessation. Bacterial infections are a common sequel to viral respiratory infections; this might have been the case here, or the infections could have been co-incidental. Renal disease is common in cats, and Cat 4A’s Stage 2 disease could also have been a co-incidental rather than related event. However, research has shown that humans are at an increased risk of acute kidney injury and chronic disease following SARS-CoV-2 infection, even if they were not hospitalised [56], and this cat’s renal disease might therefore also be related to SARS-CoV-2 infection.

It can be difficult to demonstrate causal links between viral infection and observed clinical signs, particularly when the aetiopathogenesis of a viral disease is not yet fully understood, clinical presentations are diverse and potentially informative diagnostic materials such as tissue samples are not available from field cases. While associative links might be established using epidemiological data, this approach requires large numbers of cases and extensive testing for infection. Our findings suggest that more widespread testing of cats for SARS-CoV-2 infection would likely provide useful insights to the range of clinical signs associated with infection and determine where causal links exist. Continuing circulation of SARS-CoV-2 and the emergence of new variants in humans provide ongoing opportunities for reverse zoonosis. With the evidence published to date showing that different variants have different levels of infectivity and virulence in cats as well as people, ongoing monitoring of the feline population should be considered an essential aspect of a comprehensive SARS-CoV-2 surveillance programme.

## 5. Conclusions

This study demonstrated a low prevalence of RT-qPCR positivity in samples submitted for diagnostic testing from UK cats, and an unexpected dearth of cases after the emergence of the Omicron variant, despite its high infectivity in humans. The SARS-CoV-2 infected cats that were identified displayed a range of clinical signs that included mild respiratory disease, pyrexia, sudden death, chronic lip licking, chronic anorexia, respiratory distress and chronic renal disease.

## Figures and Tables

**Table 1:** Case details of cats infected with SARS-CoV-2 identified in this study.

Animal ID	Household Case	Sampling Date	Clinical Signs	Surveillance Method	RT-qPCR	Serology (PVNT)	Viral Sequence	Highest titre	GISAID Accession Number
Cat 1A	1	Feb-21	Pyrexia	Passive	+	+	Alpha	Alpha	EPI_ISL_17971924
Cat 1B	1	May-21	None, same household as Cat 1A	Passive	Not done	+		Alpha	
Cat Y		Sep-21	Mild respiratory	Passive	+	Not done	Incomplete		
Cat 2A	2	Oct-21	Mild respiratory then sudden death	Passive	+	Not done	Delta AY.4		EPI_ISL_17971926
Cat 2B	2	Nov-21	Mild respiratory, lip licking, same household as Cat 2A	Passive	Not done	+		Delta	
Cat X		Dec-21	Mild respiratory	Active	+	Not done	Delta AY.4.2.1		EPI_ISL_17971927
Cat 3A	3	Dec-21	Mild respiratory, prolonged hyporexia	Passive	Not done	+		Delta	
Cat 4A	4	Sep-22	Severe respiratory	Passive	-	+		Omicron	

**Table 2:** Medication used in the management of Cat 3A.

Drug	Trade Name	Route of Administration	Dose	Dosing Interval	Days of Hospitalisation
Maropitant	Vetemex (Virbac, Bury St Edmunds, UK)	Intravenous/subcutaneous	1 mg/kg	24h	1 to 5
Metoclopramide	Vomend (Dechra, Northwich, UK)	Subcutaneous	0.5 mg/kg	12h	5 to 7 and post-discharge
Omeprazole		Intravenous	1 mg/kg	12h	1 and 2
Mirtazapine		Oral	1.9–3.8 mg	48h	Throughout
Vitamin B		Subcutaneous	0.02 mg/kg	Once	2
Buprenorphine		Intravenous/subcutaneous/oral	0.01–0.02 mg/kg	6–8 h	2, 3, 5, 6 and post-discharge
Meloxicam	Loxicam (Norbrook, Newry, UK)	Subcutaneous	0.1 mg/kg	Once	5
Amoxycillin-clavulanate	Synulox (Zoetis, Leatherhead, UK)	Oral	100 mg	12h	Post-discharge (for 5 days)

## Data Availability

Data are contained within the article or appendices or are signposted (e.g., GISAID accession numbers) within the article.

## Supplementary Materials

Sample metadata for the seropositive samples, for which the results are given in Section 3.3, can be downloaded at: https://www.mdpi.com/article/10.3390/v15081769/s1.

## Appendix A. Supplementary Methods

### Appendix A.1. SARS-CoV-2 RT-qPCR

#### Appendix A.1.1. Reagents

PCR kit: NEB (Ipswich, MA, US) Luna^®^ Universal Probe One-Step RT-qPCR Kit E3006

Reaction mix: IDT (Coralville, Iowa, US) 2019-nCoV RUO Kit #10006713

PCR positive control plasmid: IDT 2019-nCoV_N_Positive Control #10006625

Primers/probes: IDT CDC panel #10006713

#### Appendix A.1.2. Primer/Probe Sequences

SARS-CoV-2 N1 forward: GACCCCAAAATCAGCGAAAT

SARS-CoV-2 N1 reverse: TCTGGTTACTGCCAGTTGAATCTG

SARS-CoV-2 N1 probe: ACCCCGCATTACGTTTGGTGGACC (label: FAM/BHQ1)

SARS-CoV-2 N2 forward: TTACAAACATTGGCCGCAAA

SARS-CoV-2 N2 reverse: GCGCGACATTCCGAAGAA

SARS-CoV-2 N2 probe: ACAATTTGCCCCCAGCGCTTCAG (label: FAM/BHQ1)

PCR machine: Applied Biosystems 7500 Real time PCR System (Thermo Fisher, Waltham, MA, USA)

**Table A1:** RT-qPCR programme for SARS-CoV-2 RT-qPCR N1 and N2 assays.

Temp (°C)	Time	Cycles
55	10 min	1
95	1 min	1
95	10 s	45
58	1 min	

### Appendix A.2. SARS-CoV-2 Viral Full Genome Sequencing

#### Appendix A.2.1. Cats 1A, 2A and Y

Sequencing libraries were prepared according to the ARTIC network nCoV-2019 (https://artic.network/ncov-2019, accessed on 14 May 2021) using sequencing protocol V.3. V3 of the ARTIC amplicon primers was used for Cat 1A, whilst V4 was used for Cats 2A and 2B. For the very low viral load sample (Cat Y) we included a mild DNaseI (Invitrogen, Waltham, MA, USA) treatment and used SuperScript IV (Invitrogen) to generate cDNA prior to amplicon generation.

Libraries were sequenced in a R9.4.1 flow cell (Oxford Nanopore Technologies, Oxford, UK) using MinKNOW version 21.05.25, raw FAST5 files were base called using Guppy. For Cat 1A Guppy version 4.0.11 was used with high-accuracy mode and a minimum quality score of 7, whilst for Cats 2A and Y version 5.0.16 was used with super-accurate mode and a minimum quality score of 10; in both cases, barcodes were required at both ends for demultiplexing.

Nanopore reads were processed using the ARTIC network artic-ncov2019 bioinformatics protocol and conda environment (https://github.com/artic-network/artic-ncov2019, accessed on 14 May 2021). Briefly, reads are size filtered, mapped against the reference strain Wuhan-Hu-1 (MN908947), amplicon primer trimmed, variant called with Nanopolish, followed by consensus sequence generation.

#### Appendix A.2.2. Cat X

Nucleic acid was converted to cDNA using LunaScript RT (NEB) and then amplicon generation and library preparation performed in a single step using the EasySeq RC-PCR SARS-CoV-2 whole genome sequencing method (Nimagen, Nijmegen, Netherlands). Libraries were sequenced on a NextSeq500 sequencer (Illumina, San Diego, CA, US) with a High output cartridge and paired end sequencing (151 × 151 cycles) using NextSeq Control Software 4.0.1.41. Raw Bcl files were converted to fastq using Bcl-Convert Version 3.9.3. Reads were then adapter and quality trimmed with trim_galore (quality 30, length 50), aligned to the Wuhan-Hu-1 reference sequence (MN908947) using bwa (version 0.7.17-r1188) [57], amplicon primer trimmed and consensus called with iVar (version 1.4.2) [58] using the V4.02 Nimagen primer BED file. For all sequences, the pangolin tool [44] was used to determine the SARS-CoV-2 lineage.

### Appendix A.3. Phylogenetic and Mutation Analysis of Cat Sequences

Each of the three feline SARS-CoV-2 genome sequences were uploaded to the UShER (Ultrafast Sample placement of Existing tRee) [59] web server (https://genome.ucsc.edu/cgi-bin/hgPhyloPlace, accessed on 7 July 2023) to analyse the sequence neighbourhood surrounding each cat’s sample. For each feline SARS-CoV-2 genome sequence, mutations with respect to the Wuhan-Hu-1 reference sequence were compared to those from the surrounding human population.

## Appendix B. Supplementary Information

**Table A2:** RT-qPCR results for Cat 2A.

Swab	N1 Assay Ct	N2 Assay Ct
Nasal	18	17.1
Oral	Undet.	Undet.
Tracheal	18.9	18.1
Rectal	Undet.	37.5

Phylogenetic subtrees for Cats 1A, 2A and X are presented in Figure A1, Figure A2 and Figure A3. Cat Y only yielded sequence reads from one amplicon in ORF1ab, with no mutations present with respect to the reference sequence, and therefore a consensus sequence could not be generated, nor the lineage determined.

**Table A3:** Spike mutations present in each feline SARS-CoV-2 genome sequence with respect to the ancestral Wuhan-Hu-1 reference sequence. The synonymous mutation L821L (highlighted in red) was the only mutation not seen in phylogenetically close human SARS-CoV-2 sequences.

Cat X (Delta AY.4.2.1)	Cat 2A (Delta AY.4)	Cat 1A (Alpha B.1.1.7)
T19R	T19R	
V36F		
T95I	T95I	
		I68I
		H69del
		V70del
G142D	G142D	
		Y144del
Y145H		
E156del	E156del	
F157del	F157del	
R158G	R158G	
A222V		
L452R	L452R	
T478K	T478K	
		N501Y
		A570D
D614G	D614G	D614G
P681R	P681R	
		P681H
		T716I
		L821L
D950N	D950N	
		S982A
		D1118H
	L1141F	
